# Sleep, Cognition and Cortisol in Addison’s Disease: A Mechanistic Relationship

**DOI:** 10.3389/fendo.2021.694046

**Published:** 2021-08-27

**Authors:** Michelle Henry, Kevin Garth Flusk Thomas, Ian Louis Ross

**Affiliations:** ^1^Centre for Higher Education Development, University of Cape Town, Cape Town, South Africa; ^2^ACSENT Laboratory, Department of Psychology, University of Cape Town, Cape Town, South Africa; ^3^Division of Endocrinology, Department of Medicine, University of Cape Town, Cape Town, South Africa

**Keywords:** Addison’s disease, cortisol, sleep, cognition, circadian rhythm

## Abstract

Sleep is a critical biological process, essential for cognitive well-being. Neuroscientific literature suggests there are mechanistic relations between sleep disruption and memory deficits, and that varying concentrations of cortisol may play an important role in mediating those relations. Patients with Addison’s disease (AD) experience consistent and predictable periods of sub- and supra-physiological cortisol concentrations due to lifelong glucocorticoid replacement therapy, and they frequently report disrupted sleep and impaired memory. These disruptions and impairments may be related to the failure of replacement regimens to restore a normal circadian rhythm of cortisol secretion. Available data provides support for existing theoretical frameworks which postulate that in AD and other neuroendocrine, neurological, or psychiatric disorders, disrupted sleep is an important biological mechanism that underlies, at least partially, the memory impairments that patients frequently report experiencing. Given the literature linking sleep disruption and cognitive impairment in AD, future initiatives should aim to improve patients’ cognitive performance (and, indeed, their overall quality of life) by prioritizing and optimizing sleep. This review summarizes the literature on sleep and cognition in AD, and the role that cortisol concentrations play in the relationship between the two.

## Introduction

Sleep is a critical biological process, an inevitable and essential aspect of normal human physiology. Nonetheless, questions about the functions of sleep (e.g., whether it is necessary for more than simple physical and mental restoration) remained unanswered until relatively recently. Available neuroscience literature provides some of the sought-after answers, suggesting that healthy, uninterrupted sleep is vital to ensuring that, for instance, consolidation of memory traces acquired during waking hours occurs smoothly and efficiently. Of particular interest here is that cortisol appears to play a particularly important role in mediating the sleep-memory relationship, especially because of its function in maintaining the integrity of sleep architecture (1, 2).

Patients with Addison’s disease (AD) require lifelong glucocorticoid replacement therapy. However, replacement medication does not restore the natural circadian rhythm of cortisol and, despite adherence, patients experience sub/supra physiological cortisol concentrations, particularly during the night. Patients with AD report and experience both poor-quality sleep and cognitive difficulties (3–8). One possible (but as yet unexplored) explanation for the sleep disruptions and memory deficits experienced by these patients is that the periods of sub- and- supra-physiological cortisol concentrations they experience may have a specific negative impact on processes of sleep-dependent memory consolidation.

This review aims to summarize and integrate the literature on the relationships between cortisol concentrations, sleep disruption, and cognitive functioning in patients with AD. This review is needed because despite expanding scientific evidence suggesting that sleep is a critical biological process and its functional value extends well beyond simple physical and mental restoration, very few published studies have investigated whether disrupted sleep is a possible mechanism that underlies the memory deficits experienced by patients with AD.

## A Brief Overview of Human Sleep

Human sleep is a natural state of reduced responsiveness accompanied by a partial loss of consciousness. Sleep is regulated by three different processes: the homeostatic process, which determines its need, the circadian process that influences its timing, and the ultradian process that determines its organization (9, 10).

Sleep is cyclical in nature, alternating between 4 and 6 repeated cycles of rapid eye movement (REM) and non-rapid eye movement (NREM) sleep, with each cycle lasting approximately 90–120 120 minutes (11–13). Human sleep patterns have some predictable characteristics. Sleep onset is characterized by rhythmic alpha waves, occurring particularly in the occipital regions as discerned by electroencephalogram (EEG). Sleep then follows with NREM (Stages 1-4 (N1-N4) before the first episode of REM. The first sleep cycle usually begins with Stage 1 (N1), which lasts for 1-7 minutes after sleep onset. Stage 2 (N2), which lasts for 10-25 minutes, is signaled by K-complexes. As N2 progresses, high-voltage slow-waves appear, signaling the start of SWS. Within SWS, Stage 3 (N3) lasts only a few minutes, whereas Stage 4 (N4) lasts for 20-40 minutes. The body may re-enter lighter stages of sleep (N1-N3) for approximately 5 minutes, before the first REM episode is initiated. This episode is short-lived, lasting only 1-5 minutes. Thereafter, NREM and REM continue to alternate in a cyclic manner throughout the night, with REM cycles becoming longer and slow-wave sleep (SWS) shorter as the night progresses. Brief waking episodes occur in the later night, usually near the transitions into REM sleep (13).

## Circadian Rhythmicity – Control of Hormone Release

The release of nearly all hormones follows daily oscillations, which result from an interaction between 24-hour circadian rhythmicity and the sleep-wake cycle (9, 14–16). Circadian rhythms are generated by the suprachiasmatic nucleus (SCN) hypothalamus, by light, and by ultradian rhythms (17, 18). The internal master clock in the SCN ensures we anticipate and prepare for changes in our environment and act appropriately (19, 20). These oscillators are synchronized with each other by the SCN’s master clock (21). Regarding the circadian clock, its timing mechanism is located in the SCN and incorporates three different components: (a) input pathways that transmit light and other environmental signals to the clock, (b) an endogenous pacemaker that generates 24-hour rhythms, and (c) output pathways that project to other brain regions and peripheral organs (9). The circadian clock is responsible for daily variations in body temperature, melatonin, and cortisol secretion, and will align those rhythms with those of sleep and other physiological processes (22). Circadian oscillators are also located in numerous peripheral tissues, including the liver, lungs, heart, and adrenal glands. These oscillators are synchronized with each other by the master clock (21). The hypothalamic-pituitary-adrenal (HPA) axis plays an important role in the homeostatic processes of the body, the co-ordination of the organism’s ability to adequately cope with environmental stressors and sleep regulation. This physiological system regulates the secretion of various hormones; of particular concern to this review is the release of cortisol resulting from HPA-axis activity.

In healthy people, cortisol has a robust diurnal secretory pattern. The highest concentrations occur in the early hours of the morning, with a peak just after waking. Concentrations then decrease slowly throughout the day, with troughs in the mid-afternoon and at midnight; the daily nadir typically happens several hours after initiation of nocturnal sleep. Concentrations then begin to rise from 02h00 to 03h00 and continue to rise until awakening. The nocturnal rise in cortisol is thought be in response to the greater energy demands of the brain as the night ends (23–26). The daily rhythm of corticotropin-releasing hormone (CRH) and adrenocorticotropic hormone (ACTH) occur in close parallel with the daily rhythm of cortisol, all highest in the morning and reaching a nadir during around midnight (12, 25, 27). In contrast, melatonin is secreted from the pineal gland and synchronized to light from retinal input and growth hormone (GH) concentrations and are highest during early sleep, suggesting a reciprocal relationship between the HPA and hypothalamo-pituitary-somatotrophic (HPS) systems (21).

## The HPA Axis and Sleep

**Figure 1:**
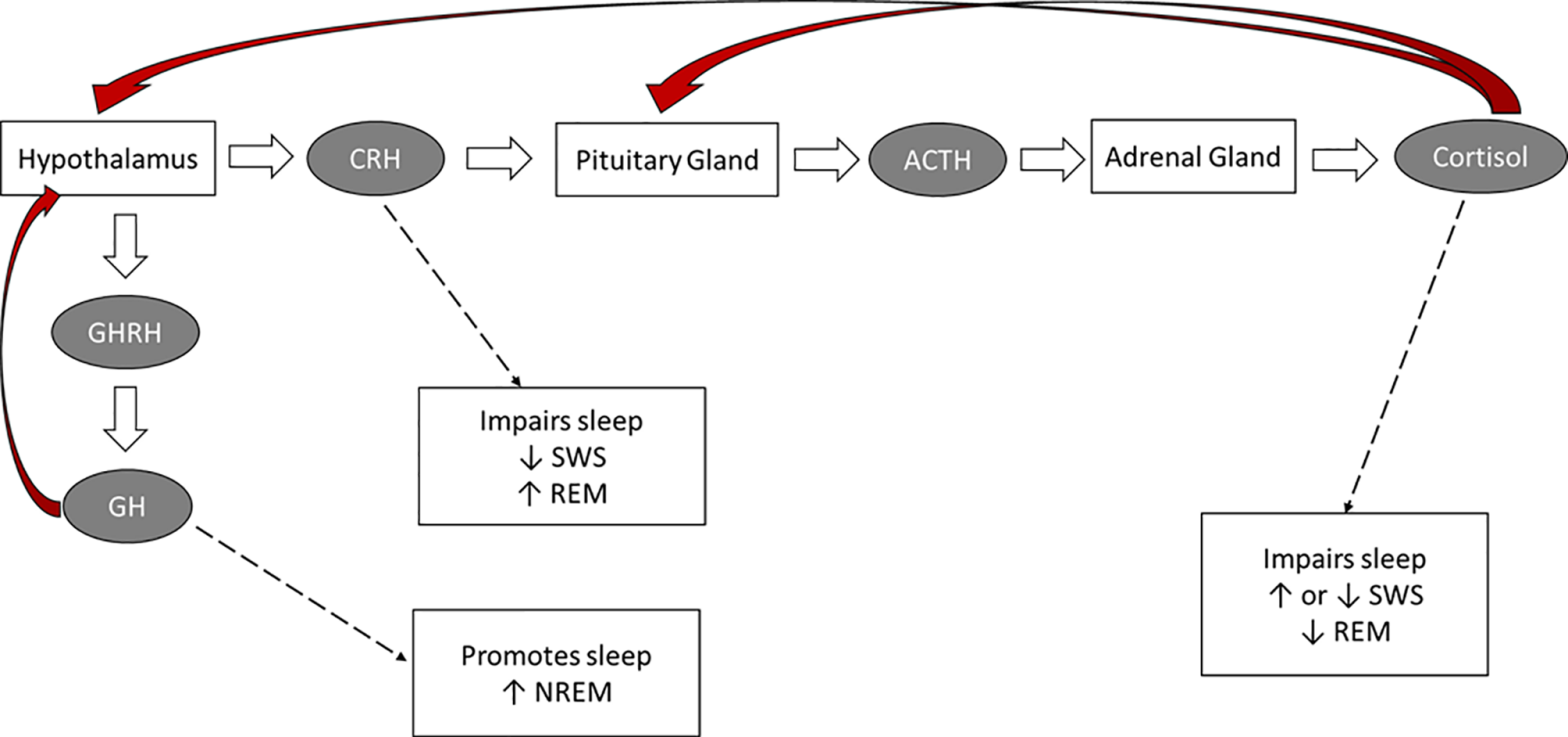
The hormonal control of sleep. Red arrows: negative feedback on the indicated brain structure.

The HPA axis plays an important role in maintaining alertness and modulating sleep [see **Figure 1**; (24, 28, 29)]. In fact, there is a bidirectional relationship between sleep architecture and HPA-axis activity. For example, cortisol exerts specific effects on sleep, whereas changes to sleep affect the release of this hormone (25, 26, 30).

### Endogenous HPA Hormones and Their Effect on Sleep

The circadian rhythmicity of specific hormones (e.g., cortisol, ACTH, CRH, GH releasing hormone, melatonin) plays an essential role in sleep timing and offset and in the distribution of sleep stages across the night (28, 31–33). Inhibitory HPA-axis actions, particularly during SWS, are responsible for attenuated cortisol activity during the first half the night. The quiescent period of HPA-axis activity starts prior to sleep, and continues into the first half the night, when SWS occurs at a maximum. Cortisol concentrations decrease rapidly in the first 20 minutes after SWS onset, and there is a consistent inverse temporal relationship between low cortisol concentrations and high SWS (15, 24, 34–37). The optimal cortisol levels during early sleep augments SWS *via* feedback inhibition of CRH (28, 33). In the second half of the night, when REM sleep predominates, inhibitory mechanisms are attenuated and HPA secretory activity slowly increases (15, 38). Cortisol, CRH, and ACTH secretion and SNS activity increase during the latter part of the night. During the last sleep cycle, increases in cortisol are paired with increases in REM (39). In summary, while the deepening of sleep during SWS is associated with decreasing cortisol concentrations and decreased sympathetic tone, high autonomic and high cortisol activity occur during REM cycles (25, 33).

The reciprocal relationship between growth-hormone releasing hormone (GHRH) and CRH also plays an important role in regulating sleep. GHRH inhibits HPA-axis activity during early sleep, stimulating NREM and promoting sleep, whereas CRH inhibits SWS, enhances REM and vigilance, and disrupts sleep (27, 40, 41). This pattern points to a reciprocal interaction between sleep architecture and hormones of the hypothalamic-pituitary-somatotrophic (HPS) and HPA systems. Confirming the sleep-promoting role of GHRH and the sleep-disrupting effect of CRH, in older adults the typical age-related reduction in GH levels is accompanied by reduced SWS, whereas in both older adults and in depressed younger adults increased CRH levels contribute to the typically-observed sleep disruptions (27, 42).

Finally, ACTH and melatonin also play a role in sleep regulation. ACTH is the prime stimulus for cortisol release during sleep, and primarily affects sleep through its impact on cortisol secretion (29, 35). The secretion of melatonin, which has a sleep-promoting effect, is dependent on the light-dark cycle and is maximal during sleep periods (43). In fact, melatonin can induce sleep even when there is an insufficient homeostatic drive to sleep. Hence, melatonin administration has been used to treat insomnia and circadian rhythm disorders as it can preclude the drive for wakefulness and produce shifts in the circadian clock so that sleep occurs at a desired time (44).

### Effects of Sleep on HPA Hormones

Sleep appears to have a direct impact on cortisol secretion. Specifically, sleep onset is associated with inhibitory effects on cortisol secretion. These effects persist for 1–2 hours after sleep onset (34, 45). In contrast, awakenings and the end of sleep are accompanied by cortisol increases (29, 46). Nocturnal awakenings are associated with releases of cortisol and subsequent inhibition of cortisol secretion (34, 40, 46).

Nocturnal awakenings and final morning awakening elicit a rapid increase in both ACTH and cortisol. Unlike nocturnal awakenings, this cortisol awakening response (CAR), includes a 50–60% increase in cortisol secretion, lasting an hour, with a peak at about 30 minutes after awakening (47–50). Some research suggests that the release of ACTH and cortisol during late sleep is precipitated by the physiological expectation that sleep will end at a certain time, and/or by the anticipation of the stress of waking (51–53).

### Effects of Sleep Disruption on Circadian Rhythms

Acute shifts in the sleep-wake cycle (such as daytime sleeping or napping or the consequences of jetlag and shift-work), reduced sleep quality, and sleep deprivation all lead to HPA-axis activation and hence can alter the normal circadian pattern of cortisol secretion (34, 54–59).

Regarding poor sleep quality, its experience (and, in fact, even its mere perception) is associated with increases in basal cortisol levels. Such increases stimulate arousal and suppress sleepiness, thus increasing sleep disturbances [which, in empirical studies, are characterised by increased wake time and reduced REM sleep; (16, 24, 60, 61)].

Regarding sleep deprivation, several studies report that elevated cortisol concentrations are present during both the sleep deprivation period and the subsequent day and evening (33, 59, 62–66). Some researchers explain this physiological pattern by speculating that the initial sleep deprivation period activates the HPA axis as part of the stress response and may also reflect a decrease in the negative feedback regulation of the HPA axis. Thereafter, prolonged wakefulness increases sleep pressure (the increased need to sleep after periods of wakefulness), leads to fatigue and sleepiness, and causes a blunting of HPA-axis activity (34).

However, sleep disruptions do more than just impact the circadian rhythm of HPA-axis hormones. Disrupted sleep has detrimental effects on health, quality of life, mood and cognition, which is not surprising given the central role of sleep in physiological restorative processes, emotion regulation and memory consolidation (1, 10, 24, 67).

## Sleep and Memory

The ability to effectively remember relies upon three broad cognitive processes: encoding (the transformation of new information into a form that can be stored in memory), consolidation (the stabilization of new memories in the brain), and retrieval (68, 69). One of the most important ways in which sleep affects cognition is by helping to consolidate memories (70–72). The process of *memory consolidation* involves strengthening of memory traces, which represent information of our experiences, and the parallel integration of these experiences with previously acquired knowledge (69, 73).

**Figure 2:**
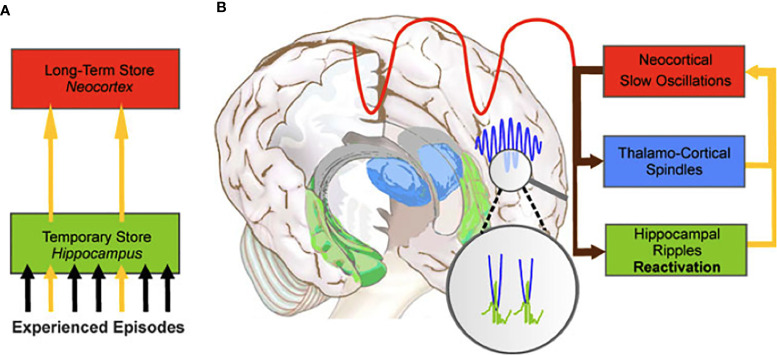
The hippocampal-to-neocortical dialogue. **(A)** During NREM sleep, memories temporarily stored in the hippocampus are transferred to the long-term store in the neocortex. **(B)** The dialogue involves the interaction between the slow oscillations, sleep spindles and hippocampal ripples to create *spindle-ripple events* (magnified circle). From Born et al. (75). System consolidation of memory during sleep. *Psychological Research, 76*, 192-203.

Whereas encoding of environmental events (i.e., acquisition of information) and retrieval of those memories (i.e., reconstruction of previously acquired information) takes place during waking hours, the process of memory consolidation is incompatible with waking consciousness (1). Hence, when the organism effectively loses consciousness for several hours during sleep, physiological conditions are optimal for memory consolidation to take place. This is why theories regarding the function of sleep have gradually come to accept that a central aspect of this stage of consciousness is to strengthen memories encoded during waking and to subsequently transfer their traces into long-term storage (1, 74). Sleep-dependent memory consolidation (see **Figure 2**) appears to involve (a) repeated reactivation of information encoded during waking, and (b) transformation of newly acquired unstable memories into stable representations that become integrated into existing knowledge networks, thus forming long-term memories. In other words, during sleep the organism experiences “off-line” periods (i.e., periods that do not feature the kinds of interference experienced during waking) during which newly encoded memories are transferred from temporary to long-term stores (75, 76). The details of how these steps are accomplished, and which neural regions and neurobiological processes support them, remains somewhat controversial, however [for reviews, see (1, 2, 77–79)].

Evidence for sleep-dependent memory consolidation is provided by numerous studies indicating that sleep enhances retention of information learned during waking hours, and that a sleep-filled delay enhances performance on a variety of declarative and non-declarative memory tasks (2, 80–89). In contrast, when sleep is disrupted memory performance is poorer than when individuals are allowed to sleep uninterrupted (90–92).

## Cortisol: A Functional Role in Memory

Adequate concentrations of cortisol are essential for optimal cognitive functioning (93–97). The hippocampus plays a vital role in memory consolidation and in new learning, encoding, and retrieval of declarative memories (97–105), while the prefrontal cortex (PFC) is similarly important for integrating sensory information, evaluating the significance of environmental stimuli, and processing previously encoded materials (106–110). Because both these structures contain particularly high concentrations of glucocorticoid receptors (111), any alterations in cortisol secretion have marked effects on their functioning. A substantial body of data indicates that elevated cortisol concentrations negatively impact performance on hippocampal-dependent memory tasks [e.g., word list and paragraph recall tasks; (94, 112–120)] and on PFC-dependent working memory and executive functioning tasks [e.g., tests assessing set-shifting, attention, abstract thinking, cognitive flexibility, mental rotation; (120–129)].

Studies have consistently demonstrated that chronically elevated glucocorticoids impair hippocampal-dependent memory (130–134)^1^. The negative impact of elevated cortisol on verbal declarative memory performance has been demonstrated (a) following increases in endogenous levels of the hormone (through laboratory-based stress induction procedures; 127, 139, 141–143), and (b) in studies that featured exogenously administered corticosteroids (128, 144–148). For example, Kirschbaum et al. (94) found that stress-induced cortisol increases, and (separately) administration of 10 mg hydrocortisone orally, were associated with poorer recall of verbal material. Similarly, De Quervain and colleagues (149, 150) found that oral administration of 25mg of cortisone acetate significantly impaired both free and cued recall of verbal material, while leaving recognition memory (which is not dependent on hippocampal substrates) unaffected, and that the same dose of cortisone acetate impaired cued recall of a series of word pairs. In that study, stress-level doses of cortisone acetate reduced cerebral blood flow to the medial temporal lobe (MTL), a memory network that broadly includes the hippocampus. Several studies have also documented impaired performance on spatial memory and navigation tasks in the presence of elevated cortisol levels in humans (94, 127, 146, 151–162). However, investigations of the impact of cortisol on spatial memory are more abundant in the animal literature. Furthermore, studies investigating spatial memory and cortisol in humans have produced more variable results, compared to the robust literature on impaired verbal memory in the presence of elevated cortisol levels.

Although hippocampal-dependent forms of memory are impaired by increased cortisol concentrations, non-hippocampal forms of memory (e.g., procedural memory), appear unaffected (e.g., 117, 163, 164). For example, Kirschbaum and colleagues (94) found that oral administration of 10mg cortisol to healthy subjects impaired performance on a declarative (a word list) but not a procedural (a word priming test) memory task. Furthermore, increased cortisol levels impair verbal declarative memory whereas non-verbal memory appears unaffected (165, 166). For example, Newcomer and colleagues (146) found that a 4-day period of oral administration of cortisol to young adults impaired their performance on a paragraph recall task, but did not significantly affect their recall of previously presented geometric line drawings or their performance on a spatial location task.

Regarding the PFC’s involvement in memory processing, this brain structure plays an important role in the encoding and retrieval of declarative memories (167). Specifically, after retrieval, the PFC determines whether an event occurred in a particular setting (168, 169), allowing accurate memories to be reconstructed. The PFC is also involved in working memory (WM). Specifically, it allows humans to (a) keep a mental “sketch” of information and protect this information from internal and external distractions, (b) inhibit inappropriate responses and behaviour, and (c) regulate attention. As such, the PFC allows for cognitive flexibility and goal-directed behaviour (121, 122).

Chronically elevated cortisol concentrations lead to dendritic atrophy in the PFC (170), and stengthens the noradrenalin system, which reduces neuronal firing within the structure (122, 171). Stress-induced cortisol increases also increase dopaminergic activity and glutamate levels in the PFC (129, 172). Glutamate receptor-mediated synaptic transmission in the PFC is particularly important for WM (173, 174). While acute elevations in glutamate have a positive effect on WM (129), excessive elevations cause impairment. Each of these hormones (noradrenalin, dopamine and glutamate) has an inverted-U influence on WM, with either too little or much impairing PFC functioning (122).

### Glucocorticoid Receptors and Memory

Glucocorticoids affect the human brain by their interaction with two intracellular receptors (134). Glucocorticoids that enter the brain change gene expression by binding to type 1 mineralocorticoid receptors (MRs) and type 2 glucocorticoid receptors (GRs). These two receptors bind cortisol with different affinities (135). MRs have a high affinity for cortisol and become heavily occupied at low cortisol concentrations (including the evening nadir of the cortisol circadian profile, when 90% of MRs, but only 10% of GRs, are occupied). In contrast, GRs have a lower affinity for cortisol and only become heavily occupied when cortisol levels reach a peak [e.g., after a stressor or after the post-awakening cortisol surge; (97, 134, 146, 175)].

MRs are found predominantly in the hippocampus, whereas GRs are distributed throughout the brain. Both play important roles in cognitive function, however (176, 177). MRs are located in brain regions involved in behavioral reactivity to new events, which enables the encoding of new information and subsequent retrieval, whereas GRs are located in brain regions involved in the consolidation and storage of information learned (94, 132, 178, 179). Hence, the activation of both receptors is a necessary for optimal memory functioning. For instance, de Kloet et al. (135) showed that when cortisol levels were mildly elevated (and therefore all MRs, but only some GRs, were activated), long-term-potentiation (LTP; the reinforcement of synaptic connections necessary for information storage) was enhanced. However, at higher cortisol levels (when GRs were over-activated and MR occupation was low), LTP was impaired. MRs play a particularly important role in hippocampal-dependent memory, executive function, and attention (126, 180–184). In confirmation of the latter, Schultebraucks and colleagues (185) found that, during high MR occupation, verbal memory was significantly better and there were trends towards better executive functioning.

### Variations in Cortisol Concentrations and Their Effects on Cognition

Most studies have focused on the deleterious effects of *elevated* cortisol levels on cognitive functioning. The negative effects of supra-physiological cortisol levels on brain structure and cognitive functioning is well known and evident in both healthy individuals and in patients known to experience chronically elevated cortisol levels [e.g., Cushing’s syndrome, depression, Alzheimer’s disease; (132, 175, 186–191)].

Elevated cortisol concentrations impair cognitive function due to their effect on specific neurobiological systems. Specifically, the relationship between glucocorticoids and cognition usually follows an inverted-U shaped pattern (192–195), where cognitive functioning is enhanced by a certain concentration of cortisol (114, 196, 197) and concentrations that are either too low or too high having impairing effects (114, 115, 135, 155, 178, 197).

Given that altered cortisol secretion plays a role in the etiology of many diseases marked by cognitive impairments [e.g., Addison’s disease, Cushing’s syndrome, Alzheimer’s disease, major depressive disorder, post-traumatic stress disorder, and metabolic syndrome; (130, 181, 185, 198–202)], and given the known alterations in cortisol concentrations in patients with AD on replacement therapy, it is important to determine the physiological mechanisms by which chronically altered circadian rhythms impact cognitive functioning. One such mechanism may be through sleep, given that a bidirectional relationship exists between circadian rhythmicity and the sleep-wake cycle, and because successful memory consolidation of information learned during the day is known to rely on sleep (1, 90, 203).

## Addison’s Disease

**Figure 3:**
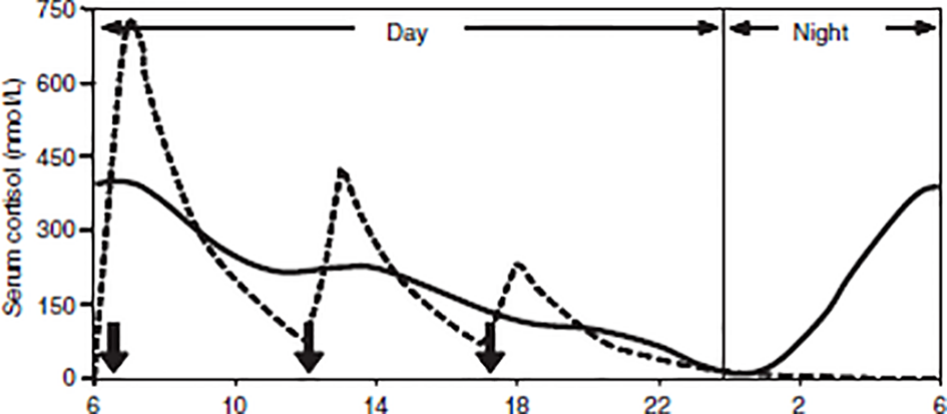
Simulated cortisol profile for a patient [broken line] following thrice-daily hydrocortisone administration [10mg at 06:00, 5mg at 12:00 and 2.5mg at 18:00, shown as solid arrows]. The normal circadian rhythm of cortisol [solid line]. From Mah et al. (211). Weight‐related dosing, timing and monitoring hydrocortisone replacement therapy in patients with adrenal insufficiency. *Clinical Endocrinology, 61(3)*, 367-375.

The diagnosis of AD is based on the measured presence of low plasma cortisol, low aldosterone levels, high renin levels, and elevated ACTH [loss of endogenous ACTH drive; (204)]. Patients with AD need to be on glucocorticoid (GC) replacement therapy for life, which is essential for survival (205). Cortisol is usually replaced with oral hydrocortisone, prednisone, or cortisone acetate (all of which activate predominantly GRs, predominantly), plus a mineralocorticoid (fludrocortisone) for sodium and potassium regulation (206, 207). Given the bidirectional relationship between cortisol and the sleep-wake cycle, the dosage, timing, and type of medication regimens used by patients may impact their general well-being and sleep patterns (28) due to the influence of GCs on circadian rhythmicity. Typically, GCs are replaced in 2-3 daily doses (see **Figure 3**), with the total daily dose ranging from 15-30mg. The highest dose (one-half to two-thirds) is taken in the morning, a reduced dose is taken in the afternoon, and (if required) a third dose in the late afternoon/evening [typically around 5pm; (208–210)]. Such a dosing schedule of GC replacement is meant to imitate the normal diurnal cortisol rhythm, to reflect the peak cortisol rise in the morning, and to avoid over-replacement in the nadir of cortisol secretion during the night (208, 211). However, despite efforts to find the best replacement regimen in terms of dosage and timing (211–213), none mimic the physiological circadian rhythm; there are still supra-physiological peaks during the day and lower-than-expected concentrations during the early hours of the morning (214–219). This over- and under-replacement results from the biochemical properties of replacement medications. Oral hydrocortisone (HC) is absorbed rapidly, reaching maximum concentrations an hour after intake (220). However, HC replacement produces extremely variable peak concentrations within a supra-physiological range, followed by rapid declines to <100 nmol/l at 5-7 hours after ingestion (221) due to its short plasma half-life (around 1.5-1.8 hours; 215, 222). This means that patients require regular dosing, and that they nonetheless experience periods of cortisol deficiency, particularly between midnight and early morning (223). Another problem with GC replacement therapy is that it does not adequately replicate the morning rise in cortisol levels experienced by healthy individuals. In healthy individuals, the natural peak of cortisol starts during the onset of REM sleep in the early hours of the morning, whereas the peak level resulting from an early morning dose of hydrocortisone comes several hours after the medication has been taken (206). This temporary early-morning cortisol insufficiency in patients with AD can account for commonly reported symptoms such as fatigue, nausea, and headaches, which are alleviated within an hour after taking the morning dose of hydrocortisone (224). Overall, conventional GCs do not restore the normal circadian rhythm of hormone release (215, 225). Instead, patients are over-replaced immediately following therapeutic administration, and then under-replaced within a few hours of that administration (211, 226), which may have important implications for sleep regulation.

### New Advancements in Treatment

As standard replacement therapy does not mimic the natural circadian rhythm, newer treatments aim to imitate physiological cortisol rhythms. These new treatments attempt to improve biochemical control of the release of cortisol and to reduce the long-term adverse effects typically associated with standard replacement regimens (220). Continuous subcutaneous HC infusion (CSHI) and modified release HC (MR-HC) tablets are two promising new treatments.

Infusion of HC in patients with AD has been shown to mimic normal circadian rhythmicity and improve quality of life (QoL; 226, 227). A crossover randomized clinical trial (*N* = 33 patients with AD who received CSHI or thrice-daily conventional therapy for a 3-month period), found that a 10mg/m^2^ daily dose of CSHI normalized cortisol and ACTH levels in the morning, and patients 24-hour cortisol curves approached normal circadian variation compared with conventional oral replacement. The 24-hour area under the curve (AUC) did not differ between infusion and conventional oral therapy, but daytime AUC (8am-midnight) was higher for oral replacement therapy, and night-time AUC (midnight-8am) was higher for CSHI. Infusion improved vitality and physical functioning (228) but did not improve sleep (except that sleep length increased, as measured by the Pittsburgh Sleep Quality Index (PSQI) and actigraphy). Another randomized double-blind placebo-controlled clinical trial (*N* = 10 patients with AD) assessed whether CSHI improved QoL and fatigue, compared to standard GC therapy (229). CSHI did not improve health status in AD patients who had mild deficits in well-being at baseline. Overall, it appears that CSHI benefits some, but not all, patients in that it restores the usual circadian cortisol rhythmicity and improves QoL (226).

**Figure 4:**
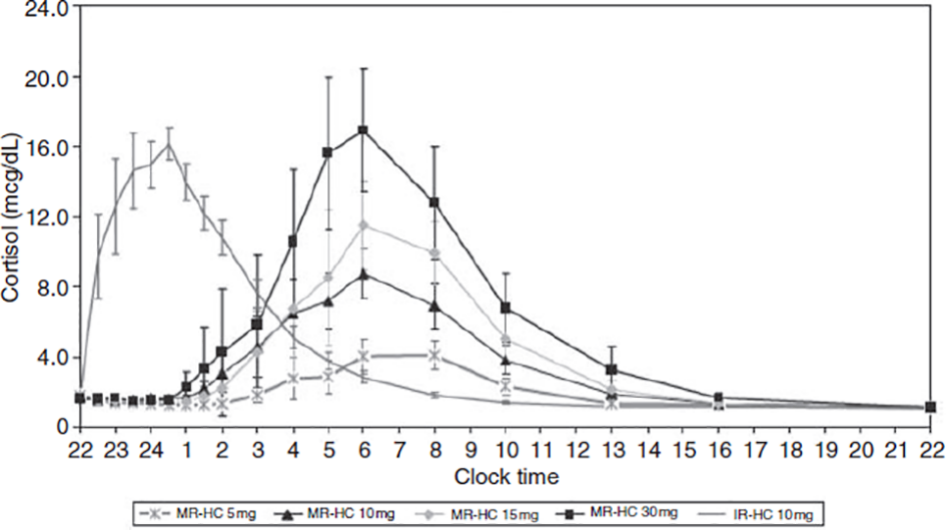
Concentration-time profiles for modified-release hydrocortisone (MR-HC) 5mg, 10mg, 15mg and 30mg compared with immediate-release hydrocortisone (IRHC). Graph showing delayed and sustained release characteristic of MR-HC (to convert values from mcg/dl to nmol/l x 27.59). From Debono et al. (230). Modified-release hydrocortisone to provide circadian cortisol profiles. *The Journal of Clinical Endocrinology & Metabolism, 94(5)*, 1548-1554.

A significant disadvantage of subcutaneous infusions is their impracticality. An alternative is for patients using HC to wake up at 3am and take a dose of medication. This alternative is perhaps even more impractical and, moreover, may cause more daytime fatigue as well as supra-physiological peaks. MR-HC offers a more practical and sustainable approach to normalizing cortisol circadian rhythms due to its immediate and extended hormone release characteristics. MR-HC has been shown to mimic natural physiological cortisol circadian rhythm (230, 231). Johannsson and colleagues (231) demonstrated that taking a once-off morning dose of either 5 or 20mg MR-HC led to a closer mimicking of physiological cortisol circadian rhythms, except for the early-morning cortisol peak. However, if MR-HC is taken late at night (thus allowing for a delayed and sustained release), it can mimic the rise in cortisol that typically occurs during the early hours of the morning. For example, Debono et al. (230) showed that taking 15-20mg of MR-HC at 23h00 and 10mg at 07h00 reproduced the normal physiological cortisol circadian rhythm in healthy controls (see **Figure 4**). In that study, participants’ cortisol concentrations peaked, on average, at 08h32 and decreased throughout the day, reaching a nadir, on average, at 00h18. Dual-release hydrocortisone (DR-HC; Plenadren) has both an immediate-release coating and extended-release core. This form of replacement therapy better mimics the normal cortisol profile (218) and improves patients’ quality of life (232–234). However, despite normalizing cortisol patterns, DR-HC has shown to have little effect on cognitive functioning or sleep (235). Although Krekeler and colleagues (235) showed that patients with adrenal insufficiency treated with DR-HC tended to show better executive functioning compared to patients on conventional HC, other cognitive domains appear unaffected, and they found no between-group differences in terms of sleep.

### Sleep Disruptions in AD

Numerous studies suggest that, in patients with AD, clinically relevant fatigue persists despite replacement therapy. For instance, Løvâs, Logeŧ, and Husebye (236) found that patients with AD self-reported reduced general health perception and vitality despite receiving replacement therapy with cortisone acetate and fludrocortisone. Similarly, van der Valk and colleagues (237) found that 48% of their patient sample (*N* = 328) self-reported abnormal fatigue; 61% reported severe fatigue. Researchers postulate that reports of increased daytime fatigue may be due to reduced quality of sleep in patients with AD and that relatively increased doses of HC may contribute to these sleep disruptions (3, 236, 238).

Løvâs, Husebye, Holsten, and Bjorvatn (3) found that 34% of the 60 patients with AD in their sample self-reported weekly sleep disturbances (difficulties falling asleep [13%], repeated awakenings [14%], and early morning awakenings [20%]). Similarly, Henry et al. (7) found that 60 patients with AD self-reported poorer sleep quality and efficiency, longer sleep latency, shorter sleep duration, more sleep disturbances, and greater daytime dysfunction compared to healthy controls. In terms of objectively measured sleep quality, Henry et al. (5) found, using actigraphy, that patients with AD experienced interrupted sleep characterized by worse sleep efficiency and a greater amount of time spent awake compared to healthy controls, who achieved a fuller night of uninterrupted sleep. These data present a pattern showing that patients with AD report frequent sleep disturbances, including difficulty falling asleep, nighttime awakenings, and a lower sleep quality. In all of the abovementioned studies, although patients were on replacement therapy, their cortisol secretion differed from healthy individuals. Because of cortisol’s key role in regulating our circadian rhythms and ensuring the transitions between sleep stages (33), it is unsurprising patients on replacement therapy still experience poor-quality sleep.

In terms of objectively measured sleep quality *via* polysomnography, limited information exists on the impact of low cortisol concentrations on sleep architecture. Similarly, few studies report on sleep in patients with AD when replacement medication is administered by conventional replacement. In terms of low cortisol concentrations, Gillin et al. (239) reported that patients with AD whose replacement medication was withheld for longer than 24 hours (and who therefore had undetectably low concentrations of cortisol at bedtime), showed increased time spent in SWS and correspondingly reduced time spent in REM sleep. Similarly, Garcia-Borreguero et al. (238) reported that patients with AD who were deprived of glucocorticoid medication for 1.5 hours prior to bedtime (and who therefore had undetectably low levels of cortisol at bedtime) showed increased wake after sleep onset (WASO) and REM latency and decreased amount of time spent in REM sleep, compared to patients who took their medication just before bedtime. These results suggest that the initiation and maintenance of REM sleep is facilitated by cortisol. In contrast, high cortisol concentrations appear to reduce the amount of time spent in SWS (27, 35, 240), and, consistent with this, relatively lower cortisol concentrations in healthy controls (administration of metyrapone) and patients with AD (replacement medication was withheld) are significantly associated with increased delta sleep (239).

Gillin et al. (239) reported that when medication was administered by conventional replacement, patients with AD had similar sleep to controls, except that patients took significantly longer to fall asleep and spent significantly more time in delta sleep (i.e., SWS). More recently, Henry and colleagues (4) found that when medication was administered by conventional replacement, patients with AD (compared to healthy matched controls) spent significantly less time in SWS and that there was a trend towards patients experiencing significantly shorter REM latency and more time in Stage 2 sleep.

Overall, few studies have objectively assessed sleep in patients with AD, despite that fact that an abundance of scientific evidence suggests that these patients experience disruptions to the cortisol circadian rhythm and, consequently, are at risk for experiencing negative effects on sleep architecture. For instance, patients with psychiatric conditions that exhibit elevated cortisol concentrations (e.g., depression and post-traumatic stress disorder) experience less time in SWS, shortened REM latency, increased REM sleep and density, and sleep discontinuity (238, 241–244). Similarly, in patients with Cushing’s disease (who produce excessive amounts of cortisol), findings show that SWS is decreased, REM latency shortened, REM density is elevated, and aberrances in sleep continuity occur (244, 245). One study found that elevated cortisol concentrations in 11 patients with Cushing’s disease were associated with lower REM activity, and more awakenings during sleep (245). Similar patterns of decreased REM latency and/or increased time spent in REM sleep have been found in patients with AD who took hydrocortisone before bedtime and in healthy controls with artificially increased cortisol concentrations (41, 238). There are comparatively few data on sub-physiological cortisol levels and sleep, and similarly, few studies on sleep of patients with AD when replacement medication is administered as part of routine replacement. Therefore, effects on sleep quality and architecture of the illness itself, and of replacement therapy, remains largely unexplored in patients with AD.

Because of the central role the HPA axis plays in sleep regulation (28, 35), either low or high night-time cortisol, alongside high night-time ACTH and CRH, may lead to sleep disturbances in patients with AD (228). However, the implications of exposure to altered circadian cortisol patterns and consequent sleep disruptions have not been adequately addressed in the available literature. For instance, disrupted sleep may impede sleep-dependent memory consolidation. Because changes in sleep and memory are associated with the use of corticosteroids, further research is necessary to help understand the impact that replacement medication used by patients with AD has on the processes that influence sleep-dependent memory consolidation.

### Cognitive Functioning in AD

Despite replacement therapy, patients with AD frequently present with both subjective cognitive complaints and objective cognitive impairments, including poor memory and impaired concentration (206, 207, 221). Due to the affinity between variations in cortisol concentrations and impaired performance on tests of memory, attention and executive functioning (115, 146, 246), understanding how these domains are affected is relevant in patients with AD. However, very few studies have characterized cognitive function in AD.

Klement et al. (247) reported that patients with AD on replacement therapy performed significantly more poorly than healthy controls on a declarative memory test. Similarly, Schultebraucks and colleagues (185) found that patients performed significantly more poorly than controls on a test of verbal learning, and Henry et al. (6) found that patients performed significantly more poorly than controls on tests of both verbal learning and memory (and that patients made significantly more false alarms [incorrectly saying a word on the list when it was not present] when recalling information). Henry et al. (5) found that healthy controls learned and retained more information than patients with AD on two different tasks of declarative memory, but that there were no significant between-group differences for procedural memory tasks. Tiemensma and colleagues (248) found that patients performed significantly more poorly on tests of both verbal and visual memory than healthy controls. The latter study also found mild executive impairment and significantly slower processing speed in their patient group. Interestingly, in this study, delaying HC intake in another group of patients with AD, which resulted in significantly lower cortisol concentrations at the time of cognitive testing, had no impact on cognitive performance. In terms of disease characteristics and cognition, Henry et al. (6) also found that patients who had AD for a longer interval had a slower speed of processing and that patients who were diagnosed later in life had poorer declarative and working memory, a slower speed of processing, and an overall greater cognitive impairment. Blacha et al. (249) found patients with AD (20 PAI and 20 SAI) showed significantly worse performance on a test of attention compared to controls (but found no difference in memory and other cognitive domains). They also found that higher HC doses impaired attention, visuo-motor skills and executive function, but that duration of therapy had no impact on cognitive performance. Similarly, Harbeck et al. (223) found that higher cortisol levels were associated with impaired short-term memory in patients who underwent short-term hydrocortisone infusion during the night. Overall, it appears that cognitive deficits in patients with AD are primarily in the domain of declarative memory (both verbal and visual memory), but also extend to executive functioning (including attention and processing speed).

Impaired declarative memory performance likely emerges from two main sources. First, indirectly, disrupted cortisol secretion patterns impact circadian rhythms and lead to sleep disturbances. Second, directly, due to supra-physiological cortisol levels experienced by patients taking short-acting hydrocortisone (250). Supra-physiological glucocorticoid increases impact on brain regions such as the hippocampus and the PFC which have a high concentration of glucocorticoid receptors (105, 251). These effects include, but are not limited to, degeneration of hippocampal neurons (252), altered organization of dendrites in the PFC (253), and, as such, impaired performance on tasks involving declarative memory (121, 254). In support of this, elevated cortisol concentrations associated with normal aging have been linked to ventricular enlargement, neuronal loss, and decreased volume in the hippocampus alongside a decline in cognitive performance (154, 252, 255–257). Exogenous administration of hydrocortisone (occurring as a once off to a few days) to healthy subjects raising serum cortisol concentrations, impairs verbal memory, working memory, visuo-spatial memory, and executive functioning (96, 146, 150, 191, 258, 259). Similarly, exogenous administration of dexamethasone or prednisone to healthy subjects impairs memory performance (117, 191, 259, 260). Prolonged levels of increased hydrocortisone may cause permanent death of hippocampal neurons, reduce hippocampal glucose uptake (255) and neuronal excitability (261), impair synaptic plasticity (262, 263), decrease the amount of newly-generated neurons, alter synaptic density in the CA1 and CA3 regions (264), and cause death of dendrites in hippocampus (252).

Another neurobiological mechanism that may explain the memory deficits observed in patients with AD is the differential activation of the two types of receptors discussed earlier, MRs and GRs. Cortisol’s effects on the hippocampus and PFC are mediated by the interaction of glucocorticoids with MRs and GRs (134). Activation of MRs is essential for successful encoding, whereas activation of GRs is essential for successful consolidation and retrieval of memory (94, 135). Activation of both receptors is required for optimal memory performance (135). In one study providing empirical support for this proposed neurobiological mechanism, Tytherleigh et al. (207) found that adequately treated AD patients, performed significantly better a declarative memory recall task, when both receptor types were activated, compared to when only one or the other was activated. While some cortisol is needed to enhance cognition (a shift towards predominant MR activation and minimal GR activation), prolonged exposure and/or high concentrations of cortisol (predominant GR activation) have deleterious effects (135, 265–267). In support of the beneficial effects of MRs on cognition, Schultebraucks et al. (185) used a repeated-measures crossover design and either administered patients with AD fludrocortisone (resulting in high MR occupation) or withheld the same drug from them (resulting in low MR occupation). Verbal memory performance was significantly better when MR occupation was high, and there also were trends towards better executive functioning in the condition.

Previous studies have shown that, in healthy adults across the lifespan, elevated cortisol levels impair cognitive functioning in ways that are predictable and that can be explained neurobiologically (105, 142, 166, 252). In AD, cortisol concentrations fluctuate –elevated far above basal levels (e.g., after hydrocortisone administration) or low [e.g., several hours after hydrocortisone medication has been taken and due to the fact that this medication has a relatively short half-life of roughly 1.5 hours; (223)]. Since the relationship between cognition and GCs usually follows an inverted-U shaped pattern, cortisol concentration variability in patients with AD may play an important role in their cognitive functioning. Furthermore, due to the known association between altered cortisol and impaired performance on standardized memory tests, between altered cortisol and disrupted sleep, and between sleep and memory consolidation, assessment of other contributors (e.g., disrupted sleep) that may contribute to deficient memory performance in patients with AD needs to be understood.

### The Relationship Between Sleep and Cognition in AD

The orderly night-time sequence and transition between SWS and REM sleep provides optimal conditions for memory consolidation (1). Consolidation begins during SWS, when specific physiological conditions (e.g., slow oscillations in neocortical networks, HPA axis suppression) allow the reactivation of memories encoded during wakefulness (268). During REM sleep, physiological conditions (e.g., suppression of norepinephrine, increased levels of acetylcholine and serotonin, ponto-geniculo-occipital and theta waves) allow reactivated memories to be integrated with pre-existing knowledge, thereby facilitating long-term potentiation (269). Cortisol’s influence on successful memory consolidation during healthy sleep is accounted for because it plays a pivotal role in sleep stage initiation and maintenance (68). Although a well-known relationship between healthy sleep and optimal memory performance is noted (270), limited studies have explored this association in patients with AD.

Henry and colleagues (7) obtained data from self-reported questionnaires and suggest that memory impairment may be mediated by sleep disruptions in AD. Henry et al. (5) investigated the relationship between adrenal function, and objectively measured sleep and cognitive performance. Results showed that periods of sleep rather than wake benefited declarative memory retention in healthy controls’ but not in patients with AD. These findings concur with a large body of literature indicating that a full night of uninterrupted sleep has positive effects on memory. Because patients with AD do not have normal circadian rhythmicity, the sequence and transitions of sleep stages may not have occurred in such a way that is required for successful memory consolidation. Another possible explanation is that patients with AD are generally fatigued, and that therefore poor performance occurs whether one sleeps or not. The design of this study did not allow consideration that patients with AD may suffer from global fatigue and therefore poor performance. Another finding in this study was that, on a story recall test, patients had greater recall when a period of wake rather than wake separated learning from recall (counterintuitive to the body of literature that sleep is an offline process beneficial for the consolidation of learned information). This result corroborates in patients with AD that sleep may not be providing an optimal period for consolidation of previously learned material. In contrast to the patterns of data on declarative memory tests, no significant between-group (AD *versus* controls) or between-condition (Sleep *versus* Wake) were found on a test of procedural memory. These results may have emerged because declarative memory tasks are hippocampal-dependent, whereas procedural tasks are not. Since hydrocortisone affects hippocampal integrity but not areas typically associated with response-based sequence learning (e.g., motor cortex, caudate nucleus), it is possible that procedural memory performance of patients with AD is unimpaired. No prior study had investigated procedural memory in patients with AD, and hence this suggestion that procedural memory is not impaired in patients with AD is a novel finding.

### The Relationship Between Sleep, Emotion Regulation, and Cognition

While it is well established that sleep plays a crucial role in various aspects of health, and cognition, sleep also plays an important role in the processing and regulation of emotion (1, 69, 79, 271). The experience of sleep deprivation or poor sleep quality makes people more sensitive to emotional and stressful events on the following day, elevates negative emotions (including feeling more irritable, angry, and anxious), and reduces positive emotions (272–275). Short sleep duration and poor-quality sleep is also associated with elevated depressive symptoms (276). REM sleep plays a particularly important role in emotion regulation (79, 277), with research showing that patients with mood disorders have altered REM sleep (278). Because of cortisols key role in sleep stage initiation and maintenance, it has an important influence on the affect regulation that takes place during healthy sleep (73). HPA hyperactivity (and consequent elevated cortisol levels, for example) plays a crucial role in the pathogenesis of medical and psychiatric disorders (e.g., major depressive disorder (MDD)) that are marked by sleep disturbances alongside mood problems (34, 279). The co-occurring presence of HPA-axis hyperactivity, sleep disturbance and mood problems in these disorders is not coincidental. Clinical studies have implied that patients with nearly all neurological and psychiatric mood disorders have co-occurring sleep abnormalities, and specifically, problems with REM sleep (79, 271). Empirical studies show that when people are deprived of REM sleep they have intensified experience of negative emotions, show increased anxiety during stressful events, and exhibit less positive reactions to positive events (275, 280).

Previously published studies in patients with AD have consistently shown that, despite being on replacement therapy, these individuals still report and experience depression and anxiety, as well as reduced stress tolerance and reduced ability to cope with daily demands (5, 236, 281). Regarding affective disorders, a Danish study of 989 patients with AD found they were 2.68 times more likely to suffer from depression than a control group with osteoarthritis (281).

Generally depressed patients self-report difficulty falling and staying asleep, and early morning awakenings; (282), and experience both SWS and REM disruptions (278). Interestingly, individuals who take hydrocortisone late at night also have decreased REM latency and increased REM sleep time, a pattern similar to that found in depressed patients. As such, altered circadian rhythms in patients with AD may explain the high presence of affective disorders in this population.

In addition to the wealth of knowledge linking sleep and affect regulation, numerous studies illustrate that affect and cognition are interrelated (283, 284), and specifically that low mood is related to impaired cognitive functioning (285, 286). The high presence of affective symptoms in patients with AD may be related to patients sleep disturbances. The high presence of affective symptoms in patients with AD may also negatively impact cognitive functioning. That is to say, the co-occurring presence of sleep disturbances, depressive symptoms, and impaired cognitive functioning in patients with AD may not be coincidental. However, no published study has explored the relationship between affect dysregulation, sleep disturbances and cognitive impairment in AD.

## Conclusion

In this article, we have summarised the current knowledge on sleep, cognition, and the association between the two in patients with AD. Numerous studies indicate that (i) healthy sleep benefits memory consolidation, (ii) alterations in cortisol activity has negative effects on sleep architecture, and (iii) sleep disruptions (e.g., as might be present in individuals with abnormal night-time cortisol concentrations) might impede the beneficial effects of sleep on memory consolidation. Sub- and supra-physiological cortisol concentrations resulting from immediate release hydrocortisone replacement therapy can have negative effects on sleep architecture and sleep-dependent memory consolidation processes. Therefore, disrupted circadian rhythms are suspected to be a major cause of sleep disturbances and cognitive impairment in patients with AD. It is well established that cortisol plays a key role in maintaining the integrity of sleep architecture and that sleep plays an important role in cognitive functioning, emphasizing the interrelationship between sleep, cognition and intact cortisol secretion.

The literature suggests that patients with AD experience disruptions to cognition, primarily in the domain of declarative memory (both verbal and visual memory), but also extending to executive functioning (specifically, attention and processing speed). Procedural memory does not appear to be impaired in these patients (although very few studies have investigated this). They also experience a reduced quality of sleep and altered sleep architecture. Hydrocortisone immediate release may lead to disrupted sleep patterns which impair optimal consolidation of learned information. Moreover, general fatigue may contribute to the presence of cognitive deficits in patients with AD. Furthermore, prolonged replacement therapy may have deleterious effects on brain regions required for optimal cognitive functioning (e.g., the hippocampus and PFC). However, studies using brain scans are needed to confirm this hypothesis.

Although prior research suggests that both cognitive and sleep complaints are frequently reported by patients with AD, only a few have used objective measures to assess either sleep patterns or memory impairments experienced by patients. Patients with AD encounter sub- and supra-physiological cortisol concentrations due to imperfect replacement therapy, ultimately altering sleep architecture and impairing cognition. It is conceivable that through modifying the pharmacokinetics of replacement therapy that these modalities in patients with AD may be improved. From a broader neuroscientific perspective, patients with AD provide a unique opportunity to simultaneously study the effects of hyper- and hypo-cortisolism on sleep quality, memory performance, and sleep-dependent memory consolidation. Careful study of these patients can help unravel the distinct roles that sub- and supra-physiological GC concentrations play in sleep regulation/structure and in sleep-dependent memory consolidation.

Although current replacement therapy aims to mimic the natural circadian rhythm of cortisol, periods of sub- and supra-physiological cortisol concentration are experienced. Both low and high cortisol concentrations can negatively impact cognition and sleep. More research is needed on the effects of dosage, duration and type of GC therapy used in patients with AD and how these impact cortisol concentrations, sleep and cognition.

Food and caffeine intake, smoking, intense exercise, and encountering stressful situations may all influence cortisol concentrations, sleep, and cognitive performance (287). Studies investigating sleep and cognition in AD should be careful to control for these potentially confounding factors. Another important contributor to cognition and sleep in patients with AD could be life-threatening events such as nocturnal hypoglycemia and adrenal crises (288). However, hardly any studies take this into account when investigating cognition and sleep. It is important to differentiate between impairments caused by the illness itself, the complications going with it or the therapy received by patients.

More studies are needed to characterize the relationship between sleep and memory, using objective measures to examine the hypothesis that poor sleep is a biological mechanism underlying memory impairment in patients with AD. More polysomnographic studies are needed to comprehensively investigate sleep architecture in patients with AD. Such studies may help explain for instance, memory consolidation in not enhanced by sleep in patients as in healthy controls. Intervention studies and clinical trials might seek to confirm this association and investigate whether the same pattern of sleep and memory deficits are present in patients, using modified-release or dual-release hydrocortisone. If it is confirmed that disrupted sleep is a vital mechanism underlying the impaired consolidation of previously learnt information, clinicians should prioritize treatment of disrupted sleep in patients with AD.

## Author Contributions

Review written by MH and IR and edited by KT. All authors contributed to the article and approved the submitted version.

## Conflict of Interest

The authors declare that the research was conducted in the absence of any commercial or financial relationships that could be construed as a potential conflict of interest.

## Publisher’s Note

All claims expressed in this article are solely those of the authors and do not necessarily represent those of their affiliated organizations, or those of the publisher, the editors and the reviewers. Any product that may be evaluated in this article, or claim that may be made by its manufacturer, is not guaranteed or endorsed by the publisher.

## Footnotes

1 On the other hand, acutely elevated GCs can either enhance (114, 134–138) or impair memory depending on several factors, including but not limited to, the time of day of cognitive testing, the stage at which a stressor is applied (i.e., at encoding, consolidation or retrieval), and the dose of GC administered (139, 140).
